# Comparative Outcomes of Pancreaticogastrostomy and Pancreaticojejunostomy Following Pancreaticoduodenectomy: A Retrospective Cohort Study from a Romanian High-Volume Center

**DOI:** 10.3390/medicina61112051

**Published:** 2025-11-17

**Authors:** Septimiu Alex Moldovan, Emil Ioan Moiș, Florin Graur, Vlad Ionuț Nechita, Luminița Furcea, Florin Zaharie, Raluca Bodea, Simona Mirel, Mihaela Ştefana Moldovan, Andreea Donca, Tudor Mocan, Andrada Seicean, Nadim Al Hajjar

**Affiliations:** 1 Department of Surgery, “Octavian Fodor” Regional Institute of Gastroenterology and Hepatology, Croitorilor Str., No. 19–21, 400162 Cluj-Napoca, Romania; septimiu1995@yahoo.com (S.A.M.); graurf@yahoo.com (F.G.); nechita.vlad@umfcluj.ro (V.I.N.); luminita.furcea@yahoo.com (L.F.); florinzaharie@yahoo.com (F.Z.); ralucabodea@yahoo.com (R.B.); cioaca_andreea@yahoo.com (A.D.); na_hajjar@yahoo.com (N.A.H.); 2 Department of Surgery, “Iuliu Hațieganu” University of Medicine and Pharmacy, Croitorilor Str., No. 19–21, 400162 Cluj-Napoca, Romania; 3 Department of Medical Informatics and Biostatistics, “Iuliu Hațieganu” University of Medicine and Pharmacy, Louis Pasteur Str., No. 6, 400349 Cluj-Napoca, Romania; 4 Department of Medical Devices, “Iuliu Hațieganu” University of Medicine and Pharmacy, Louis Pasteur Str., No. 4, 400349 Cluj-Napoca, Romania; smirel@umfcluj.ro; 5 Department of Endocrinology, County Emergency Hospital, Endocrinology Clinic, Louis Pasteur Str., No. 3-5, 400349 Cluj-Napoca, Romania; mihaelamirel@gmail.com; 6 3rd Medical Department, “Iuliu Hațieganu” University of Medicine and Pharmacy, 400162 Cluj-Napoca, Romania; mocan_tudor@yahoo.com; 7 UBBMed Department, Babeș-Bolyai University, 400349 Cluj-Napoca, Romania; 8 Department of Gastroenterology, “Octavian Fodor” Regional Institute of Gastroenterology and Hepatology, Croitorilor Str., No. 19–21, 400162 Cluj-Napoca, Romania; andradaseicean@yahoo.com; 9 Department of Gastroenterology, “Iuliu Hațieganu” University of Medicine and Pharmacy, Croitorilor Str., No. 19–21, 400162 Cluj-Napoca, Romania

**Keywords:** periampullary tumors, pancreaticoduodenectomy, Whipple procedure, pancreaticogastrostomy, pancreaticojejunostomy, postoperative pancreatic fistula, postoperative biliary fistula, postpancreatectomy hemorrhage, postoperative complications, 90-day mortality

## Abstract

*Background and Objectives*: Pancreaticogastrostomy (PG) and pancreaticojejunostomy (PJ) are the two most frequently employed reconstruction techniques following pancreaticoduodenectomy (PD), yet the optimal method remains debated. The objective of this study was to compare perioperative outcomes of PG versus PJ in patients undergoing PD for resectable periampullary tumors at a high-volume center. *Materials and Methods*: We conducted a retrospective cohort study including 604 consecutive patients who underwent PD between January 2019 and May 2025. Reconstruction of the pancreatic remnant was achieved by binding PG in 415 patients and duct-to-mucosa PJ in 189 patients. Demographics, intraoperative data, and postoperative outcomes were analyzed using standardized ISGPS/ISGLS definitions. *Results*: The overall complication rate was similar between groups (43.9% vs. 47.1%; *p* = 0.481). However, PG was associated with significantly lower rates of postoperative pancreatic fistula (POPF) (12.3% vs. 18.5%; *p* = 0.042) and postoperative biliary fistula (POBF) (2.9% vs. 6.3%; *p* = 0.044) compared with PJ. No significant differences were observed in delayed gastric emptying (DGE), postpancreatectomy hemorrhage (PPH), intra-abdominal abscess, relaparotomy, length of postoperative stay, or 90-day mortality. *Conclusions*: PG was associated with reduced rates of anastomotic fistulas compared with PJ, while other perioperative outcomes were comparable. These findings suggest that PG may be particularly advantageous in patients with a soft pancreatic remnant or nondilated duct, where the risk of fistula is higher, whereas PJ remains appropriate for firm, fibrotic glands with dilated ducts. Tailoring the reconstructive technique to pancreatic texture and ductal anatomy may therefore improve surgical outcomes and reduce postoperative morbidity.

## 1. Introduction

Pancreaticoduodenectomy (PD) is a surgical procedure primarily indicated for premalignant or malignant tumors originating from the pancreas or periampullary structures—including the common bile duct (CBD), ampulla of Vater, or duodenum—and less commonly for chronic pancreatitis or traumatic injuries [1]. In high-volume centers perioperative mortality has decreased to 4–5%, while morbidity associated with the procedure remains high, reaching up to 60%—due primarily to the complexity of the reconstruction and the high incidence of postoperative complications [2].

Among these, postoperative pancreatic fistula (POPF) is the most feared and clinically significant, with reported rates of 5–30% despite major technical progress and the adoption of standardized International Study Group of Pancreatic Surgery (ISGPS) definitions [3,4,5,6]. To reduce the risk of POPF, more than 70 modifications of the standard Whipple procedure have been described, particularly focusing on the type of pancreatico-enteric anastomosis [7]. The two main reconstruction techniques—pancreaticogastrostomy (PG) and pancreaticojejunostomy (PJ)—remain the subject of continuous debate, leaving the choice to the surgeon’s discretion—favoring the approach with which they are most comfortable and is associated with the lowest complication rates.

Reconstruction after PD involves three anastomoses: of the stomach or duodenum, the pancreatic remnant, and the common bile duct. The most debated anastomosis—having the greatest impact on postoperative outcomes—is the reconstruction of the pancreatic stump, which can be performed using either PG or PJ [5].

Various technical modifications have been developed, including duct-to-mucosa and invagination (“dunking”) anastomoses, single- or double-layer sutures, the Peng binding method, and the use of internal or external stents [8,9,10,11]. PG offers several theoretical advantages, such as a thick, well-vascularized gastric wall and exposure of pancreatic secretions to an acidic environment that may limit premature enzymatic activation, whereas PJ allows direct restoration of intestinal continuity within the same loop as the biliary anastomosis but exposes the jejunal mucosa to proteolytic injury.

Alternative pancreatic duct management strategies—such as duct ligation, occlusion with sealants, or even total pancreatectomy—have been proposed to reduce POPF risk, while the use of internal or external stents to protect the anastomosis remains debated and without clear consensus [9,12,13,14].

Several randomized controlled trials (RCTs) and meta-analyses have compared the two techniques. Some reported comparable outcomes [15,16,17,18,19], while others observed a lower incidence of POPF after PG [20,21,22,23,24]. In contrast, the RECOPANC trial found similar fistula rates but a slightly higher risk of grade A/B postpancreatectomy hemorrhage (PPH) with PG [19]. These conflicting data underline the absence of a universally accepted “best” technique, leading most surgeons to select the reconstruction method according to pancreatic texture and duct diameter—with PG favored for a soft gland and narrow duct, and PJ for a fibrotic pancreas with a dilated duct.

Additional refinements in the resection phase, such as “artery-first” approaches (posterior), uncinate-first (anterior), and other variants (e.g., mesenteric, combined), have improved the safety of PD but have not resolved the question of optimal reconstruction [25]. Furthermore, the impact of procedure variants—such as classic PD versus pylorus-preserving pancreaticoduodenectomy (PPPD)—on postoperative outcomes remains debated, as PPPD may reduce dumping and bile reflux but increase delayed gastric emptying (DGE) [26,27].

Given these ongoing controversies, there is a continuing need for large, contemporary, single-center studies applying standardized definitions to evaluate real-world outcomes of PG versus PJ. The present retrospective cohort study analyzes 604 consecutive patients undergoing PD for resectable periampullary tumors in a high-volume tertiary center. The primary endpoint of this study was the incidence of POPF, defined according to the 2016 ISGPS criteria. Secondary endpoints included the rates of postoperative biliary fistula (POBF), PPH, DGE, postpancreatectomy acute pancreatitis (PPAP), intra-abdominal abscess, relaparotomy, length of postoperative hospital stay, and 90-day mortality. By comparing these outcomes, we provided a comprehensive assessment of short-term morbidity and mortality associated with reconstruction type. The findings are intended to contribute to the ongoing debate surrounding the most effective and safest approach, particularly in high-risk patients and complex operative settings.

## 2. Materials and Methods

### 2.1. Study Design

The design of our study consisted of a monocentric retrospective cohort study, undertaken at the Surgical Department of the Regional Institute of Gastroenterology and Hepatology “Prof. Dr. Octavian Fodor”, Cluj-Napoca, Romania, between 1 January 2019 and 31 May 2025. We enrolled 604 patients who presented in our service with resectable periampullary tumors, either benign or malignant. As for the surgical treatment with curative intent, standard PD was performed in 569 patients, and PPPD was performed in the remaining 35 patients. In all patients, a standard lymphadenectomy was performed during pancreaticoduodenectomy, encompassing the peripancreatic, hepatoduodenal ligament, pancreaticoduodenal, pyloric, superior mesenteric, and celiac/hepatic artery lymph node stations, in accordance with ISGPS and National Comprehensive Cancer Network (NCCN) guidelines. The aim of our study was to establish which type of pancreatic anastomosis confers a better postoperative outcome. For that reason, we constructed two groups of patients based on pancreatic anastomosis, with 415 patients having PG and 189 patients with PJ. We must mention that in our centre, the standard technique for PG is the binding PG developed by Peng—a combination of invagination and purse-string sutures—while for PJ, we perform a duct-to-mucosa PJ, stented or non-stented. All PDs were performed by a dedicated team of senior hepatopancreatobiliary surgeons with extensive experience in PD, each having completed more than 50 such procedures prior to the study period. The same surgical team applied standardized operative steps and reconstruction techniques (binding PG or duct-to-mucosa PJ) according to institutional protocols, thereby reducing inter-surgeon variability and ensuring procedural consistency across the cohort.

The choice between the two anastomotic techniques is based on several factors, including the surgeon’s experience and preference, the consistency of the pancreatic parenchyma, and the diameter of the main pancreatic duct (Wirsung). As a general rule in our center, PG is preferred for a soft pancreatic remnant with an undilated duct, whereas PJ is typically reserved for cases involving a firm pancreatic stump, often associated with features of chronic pancreatitis and a dilated Wirsung duct (>3 mm). All cases of periampullary tumors are evaluated preoperatively by a multidisciplinary team comprising gastroenterologists, anesthesiologists, radiologists, oncologists, and surgeons, in order to determine the indication for surgery and the most appropriate operative strategy. Our institution is a high-volume pancreatic surgery center, routinely performing complex PDs, including those requiring venous resections involving the portal vein (PV), superior mesenteric vein (SMV), porto-mesenteric confluence (PV-SMV), and splenic vein (SV). In accordance with institutional standards, patients deemed suitable for radical resection were managed under the supervision of the surgical team, which maintained primary responsibility for preoperative assessment and the overall therapeutic strategy.

Comparisons between the two groups were conducted with respect to demographics, intraoperative characteristics, and postoperative outcomes.

Our work is reported by respecting the STROBE guidelines, with the STROBE checklist attached in the Supplementary Table S1: STROBE_checklist_cohort.

### 2.2. Data Collection

Patient characteristics, surgical treatment details, tumor histology, and postoperative outcomes were retrieved from our institution’s electronic database. PD was performed either as a classic Whipple procedure or as a PPPD, with PV or SMV reconstruction undertaken in cases of venous tumor involvement. Pancreatic reconstruction was achieved either by duct-to-mucosa PJ or by invaginated PG. Intraoperative variables assessed comprised estimated blood loss (mL) and operative duration (minutes). Postoperative complications were categorized as follows: infectious complications—wound infection, intra-abdominal abscess, and sepsis; fistulas—POPF, POBF, gastrojejunostomy leakage, and lymphatic leakage; other specific complications—PPH, DGE, celiac axis ischemia, and mesenteric infarction; and general complications—pulmonary complications, cardiovascular events, and *Clostridium difficile* infection. Bile culture results, when present, were classified as negative or positive. Relaparotomy, length of postoperative hospital stay, and 90-day mortality were analyzed across groups as key outcomes with significant prognostic impact.

### 2.3. Outcomes and Definitions

The overall complication rate was defined as the proportion of patients who developed at least one postoperative adverse event of any type or severity during the follow-up period. Because the institutional database lacked consistent documentation of Clavien–Dindo grading, complications could not be analyzed by severity or summarized using the Comprehensive Complication Index (CCI). Therefore, morbidity was evaluated only in terms of overall incidence, and individual complication types were reported descriptively. Infectious complications were evaluated and included: sepsis, defined as a systemic response to infection manifesting with altered temperature, abnormal leukocyte count, and evidence of organ dysfunction; intra-abdominal abscess, characterized by a localized infected fluid collection confirmed either radiologically or during reoperation; and surgical site infection (SSI), identified by erythema, purulent discharge, or a positive wound culture necessitating therapeutic intervention [28,29]. SSI remains one of the most common and burdensome complications following major abdominal procedures, particularly in hepatopancreatobiliary (HPB) surgery, where reported rates may reach up to 30% and significantly impact postoperative recovery, hospital stay, and overall outcomes [30]. PPH was defined in accordance with the criteria of the ISGPS as any episode of bleeding after pancreatic resection, independent of its timing, anatomical site, or severity [31]. Chyle leak was defined as the detection of lymphatic fluid in postoperative abdominal drainage, characterized by a milky or opalescent aspect and confirmed by a triglyceride level ≥110 mg/dL (1.2 mmol/L) [32]. In line with International Study Group of Liver Surgery (ISGLS) criteria, a POBF was defined as bilious drainage fluid with a bilirubin concentration at least threefold higher than the corresponding serum level [33]: POPF was defined according to the 2016 ISGPS criteria. A biochemical leak (BL) was defined as any drain output with amylase content greater than three times the upper limit of normal serum amylase activity, without clinical impact or need for therapeutic intervention, and was not considered a true fistula. Clinically relevant POPF (CR-POPF) included only Grades B and C, reflecting fistulas associated with clinical symptoms, prolonged drainage, or the need for interventional or surgical management [6]. Per ISGPS criteria, DGE is characterized by impaired gastric motility after surgery, manifested by failure to resume a solid oral diet within the expected postoperative period or intolerance to oral intake requiring continued nasogastric decompression [34]. PPAP was defined as an acute inflammatory condition of the pancreatic remnant occurring within the first three postoperative days after partial pancreatic resection. The diagnosis required sustained postoperative hyperamylasemia (POH), with serum amylase exceeding the institutional upper limit of normal for at least 48 h in association with clinically relevant features and radiologic alterations consistent with pancreatitis [35].

### 2.4. Statistical Analysis

Data were analyzed using R Commander 4.0.5 (R Foundation for Statistical Computing, Vienna, Austria). Continuous variables were expressed as mean ± standard deviation (SD), while categorical variables were presented as absolute numbers and percentages.

Comparisons between groups were performed using Student’s *t*-test for normally distributed data or the Mann–Whitney U test for non-normally distributed data. Chi-square or Fisher’s exact tests were applied for categorical variables, as appropriate.

Normality of quantitative data was assessed using the Shapiro–Wilk test for small samples (n < 50) and the Kolmogorov–Smirnov test for larger samples (n > 50).

Postoperative survival was analyzed with Kaplan–Meier curves and compared between groups using the log-rank test. The Cox proportional-hazards regression model was used to estimate hazard ratios (HR) and 95% confidence intervals (CIs) for the influence of the anastomotic technique on 90-day postoperative survival.

No formal power or sample size calculation was performed, as all consecutive eligible patients treated during the study period were included.

Multivariable binary logistic regression was used to identify independent predictors of POPF and POBF among 604 patients undergoing pancreatic resection. Variables clinically relevant or significant in univariate analysis were entered into the models. For POPF (n = 86), eight predictors were included: sex, age, anastomosis type, pancreatectomy type, vascular invasion, operative time, blood loss, and bile culture. For POBF (n = 24), to avoid overfitting, only anastomosis type, age, and bile culture were analyzed. Operative time and blood loss were dichotomized by their median values (280 min, 250 mL). Model fit was assessed by the likelihood ratio χ^2^ test, Nagelkerke’s R^2^, and the AUC. Univariate odds ratios (ORs) with 95% CIs were derived from individual logistic regressions with a single predictor each. Adjusted odds ratios (aORs) with 95% CIs were obtained from the multivariable model including all variables listed. A value of *p* < 0.05 was considered statistically significant.

Data on pancreatic texture and main pancreatic duct diameter were inconsistently recorded in operative notes and perioperative charts, yielding high and differential missingness (≥60%) with patterns not missing at random. As inclusion of these variables only in a complete-case multivariable model would have restricted the analysis to a non-representative subset and introduced selection or collider bias, they were excluded from the regression models.

## 3. Results

Between 1 January 2019, and 31 May 2025, a total of 604 patients underwent PD at our institution, a high-volume tertiary referral center for HPB surgery. All procedures were performed with curative intent for malignant or benign periampullary disease, and patients with palliative resections or incomplete records were excluded. Reconstruction of the pancreatic remnant was carried out according to institutional practice and intraoperative findings. In 415 patients (68.7%), continuity was restored by PG, performed as a binding technique according to the Peng modification, while in 189 patients (31.3%) reconstruction was achieved by PJ, using a duct-to-mucosa approach with or without internal stenting. The allocation of reconstruction type was guided primarily by gland texture and duct size, with PG preferred in patients with a soft pancreatic remnant and small duct, and PJ in those with a firm gland and dilated duct. This distribution reflects both surgeon preference and established consensus on tailoring reconstruction to intraoperative pancreatic characteristics.

Postoperatively, all patients were initially monitored in the intensive care unit for at least 24–48 h. Blood tests (including liver enzymes, bilirubin, pancreatic enzymes, C-reactive protein, and complete blood count) were performed daily during the first two postoperative days and every 48 h thereafter, or more frequently if clinically indicated.

Drain management followed a standardized institutional protocol. Drains for bleeding surveillance (pelvic or perisplenic) were typically removed after 48 h if no hemorrhagic output was noted. Subcutaneous drains were maintained for 3–4 days to detect potential wound infections. Perianastomotic drains—placed in the subhepatic (hepaticojejunostomy), retroanastomotic (PG/PJ), and gastroenteroanastomotic regions—were generally kept for 5–6 days. On postoperative day 5–6, an abdominal ultrasound was routinely performed: if no fluid collections were detected then drains were removed. In cases of suspicious findings (bilious, pancreatic, or enteric content in drains), inflammatory marker elevation, or ultrasound abnormalities, a contrast-enhanced computed tomography (CT) scan (IV and oral) was obtained.

Feeding jejunostomy was routinely placed in all patients and maintained for at least 14 days. Enteral nutrition (protein-rich formulas such as Nutrison or Fresubin) was initiated on postoperative day 1, with oral intake gradually advanced from liquid and hydro-lactozaharate diet on days 1–2 to a high-protein diet from day 3 onward.

In the event of suspected DGE, a nasogastric tube was reinserted and a water-soluble contrast transit study was performed. In cases of upper gastrointestinal (GI) bleeding, emergency upper GI endoscopy with hemostasis was performed. If no postoperative complications occurred, patients were typically discharged between postoperative days 8–10.

All postoperative complications were defined according to international consensus criteria (ISGPS/ISGLS), and their adjudication was performed by the surgical team based on clinical, laboratory, and imaging findings. Outcome assessors were not formally blinded to the anastomotic technique, but the institutional management protocols were standardized and consistently applied across all cases.

### 3.1. Baseline Characteristics

A total of 604 patients underwent PD during the study period, with pancreatic reconstruction performed by PG in 415 cases (68.7%) and by PJ in 189 cases (31.3%). The mean age of the entire cohort was 62.3 ± 10.4 years, with no significant difference between the PG and PJ groups (63.6 ± 9.93 vs. 62.9 ± 9.84 years; *p* = 0.442). Sex distribution was also comparable, with males representing 58.6% of the cohort (*p* = 0.565).

Histopathological analysis revealed pancreatic adenocarcinoma as the predominant diagnosis (52.3%), followed by ampullary carcinoma (20.5%) and distal cholangiocarcinoma (10.6%). The distribution of tumor types did not differ significantly between the groups (*p* = 0.358). Regarding the type of surgical procedure, classic PD was performed in 94.2% of cases, while PPPD was more frequent in the PJ group compared to PG (9.5% vs. 4.1%; *p* = 0.008).

Vascular invasion was identified in 7.0% of patients, with similar distribution between groups (*p* = 0.992). Among those requiring vascular reconstruction, tangential suture was the most common technique, used in 90.2% of cases. Preoperative biliary drainage (PBD) was performed in 59.6% of patients, most frequently by endoscopic methods (50.3%), with no significant differences between groups (*p* = 0.701). Positive bile cultures were more frequently observed in the PJ group compared with PG (57.1% vs. 49.4%; *p* = 0.001).

Mean operative time was 282.2 ± 68.3 min and mean intraoperative blood loss was 318.0 ± 259.0 mL, with no significant differences between groups (*p* = 0.155 and *p* = 0.841, respectively). Overall, most baseline variables were comparable between groups, except for a higher proportion of pylorus-preserving procedures and positive bile cultures in the PJ subgroup.

Table 1 summarizes the analysis of these clinical variables, which was undertaken to assess baseline comparability and clinical homogeneity between the subgroups.

### 3.2. Postoperative Outcomes

The distribution of postoperative complications, together with key prognostic indicators relevant to short-term outcomes (relaparotomy, postoperative hospital stay and 90-day mortality), are summarized and graphically illustrated in Table 2.

The overall complication rate was 44.9% in the entire cohort, with no significant difference between PG (43.9%) and PJ (47.1%; *p* = 0.481). Rates of infectious complications, including wound infection, intra-abdominal abscess, sepsis, and *Clostridium difficile* colitis, were comparable between the two groups. Similarly, the incidence of pulmonary and cardiovascular complications, postoperative acute pancreatitis, lymphatic leakage, mesenteric infarction, and celiac axis ischemia did not differ significantly.

**Figure 1:**
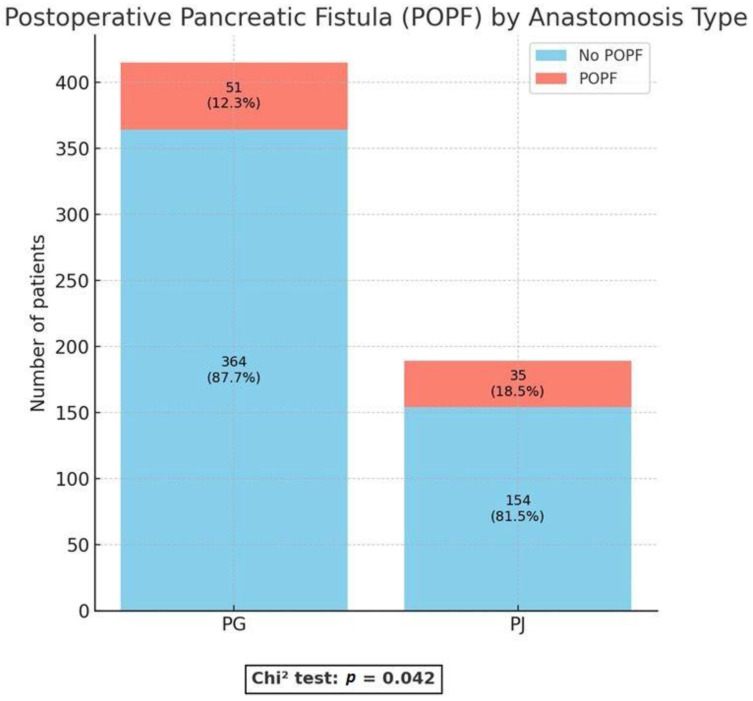
Postoperative pancreatic fistula (POPF) rate. PG = pancreaticogastrostomy; PJ = pancreaticojejunostomy.

With regard to anastomosis-related outcomes, important differences were observed. The incidence of POPF, defined as a clinically relevant fistula (Grade B/C), was significantly lower in the PG group compared with the PJ group (12.3% vs. 18.5%; *p* = 0.042) (Figure 1).

Multivariable logistic regression analysis was conducted to identify independent predictors of POPF. The model incorporated preoperative, intraoperative, and microbiological variables demonstrating clinical relevance or statistical significance in univariate analysis. Continuous intraoperative parameters were dichotomized at their median values (operative time: 280 min; blood loss: 250 mL). The final model exhibited good overall fit (χ^2^(8) = 45.6, *p* < 0.001), with a Nagelkerke R^2^ of 0.19 and an AUC of 0.77, reflecting acceptable discrimination and calibration. Model specificity was high (98.5%), whereas sensitivity was modest (13.1%), aligning with the low event rate of POPF in the cohort. aORs with 95% CIs for all covariates are presented in Table 3.

In the univariate analysis, male sex and PJ were significantly associated with the occurrence of clinically relevant POPF, whereas prolonged operative time and positive bile culture did not reach statistical significance. In contrast, the multivariable analysis, PJ, male sex, longer operative duration, and intraoperative bile contamination were independently associated with an increased risk of CR-POPF. These variables retained statistical significance after controlling for potential confounders such as patient age, tumor type, vascular resection, and intraoperative blood loss, indicating that both technical and microbiological factors contribute meaningfully to POPF occurrence.

**Figure 2:**
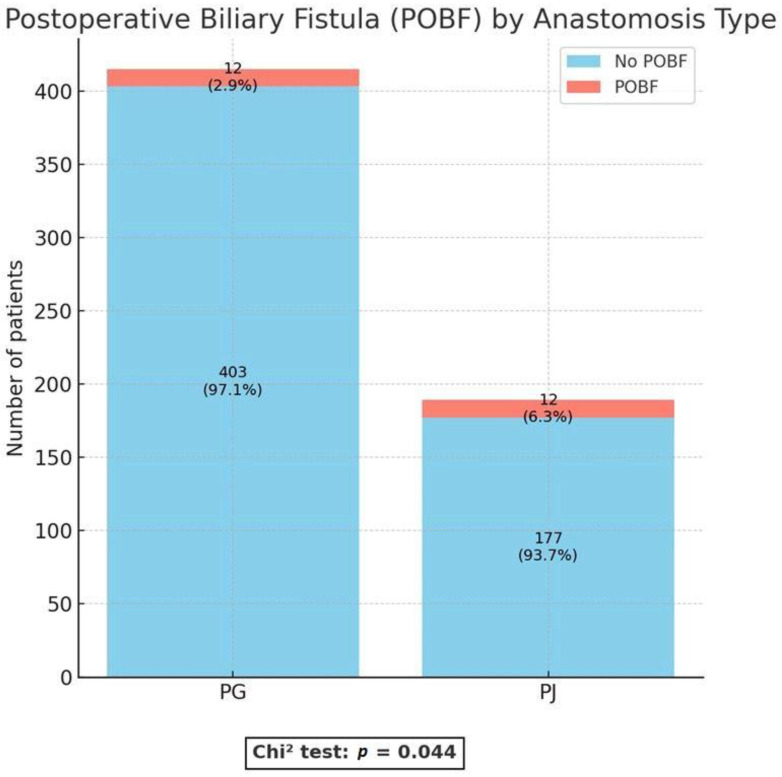
Postoperative biliary fistula (POBF). PG = pancreaticogastrostomy; PJ = pancreaticojejunostomy.

Clinically relevant postoperative biliary fistula (Grade B/C, CR-POBF) was observed significantly less often in the PG group than in the PJ group (2.9% vs. 6.3%; *p* = 0.044). (Figure 2).

A separate multivariable logistic regression analysis was conducted to identify independent predictors of POBF. Given the limited number of events (n = 24), the model was restricted to three clinically relevant variables—type of pancreatic anastomosis, patient age, and intraoperative bile culture status—to minimize the risk of overfitting. The overall model achieved statistical significance (χ^2^(3) = 10.34, *p* = 0.016; Nagelkerke R^2^ = 0.079) and demonstrated moderate discriminatory ability (AUC = 0.71). Model specificity was 100%, whereas sensitivity remained low, consistent with the rarity of POBF in this cohort.

Table 4 summarizes both univariate and multivariable logistic regression analyses. On univariate testing, only PJ was significantly associated with an increased risk of POBF, while neither age nor positive bile culture showed a significant effect. After multivariable adjustment, both anastomosis type and patient age remained independently associated with CR-POBF, supporting the robustness of these findings despite the limited event rate.

In contrast, no significant differences were noted in other specific post-pancreatectomy complications such as DGE (7.4% vs. 7.2%; *p* = 0.938) or PPH (12.7% vs. 15.7%; *p* = 0.341). Gastrojejunostomy leakage was rare and similar in both groups (2.1% vs. 0.7%; *p* = 0.138).

Regarding prognostic outcomes, relaparotomy rates (6.3% vs. 8.0%; *p* = 0.487), mean postoperative hospital stay (16.0 vs. 14.6 days; *p* = 0.135), and 90-day mortality (10.1% vs. 8.9%; *p* = 0.768) were comparable between PJ and PG groups.

Kaplan–Meier survival analysis showed no significant difference in 90-day postoperative survival between patients reconstructed by PG and those by PJ after PD. Both survival curves remained nearly superimposed throughout the observation period, with survival probabilities consistently above 95%. At 30, 60, and 90 days, the numbers of patients at risk were 388, 381, and 378 in the PG group, and 174, 172, and 170 in the PJ group, respectively. The log-rank test indicated no statistically significant difference between groups (*p* = 0.68). These results demonstrate comparable early postoperative survival for both types of pancreatic anastomosis.

**Figure 3:**
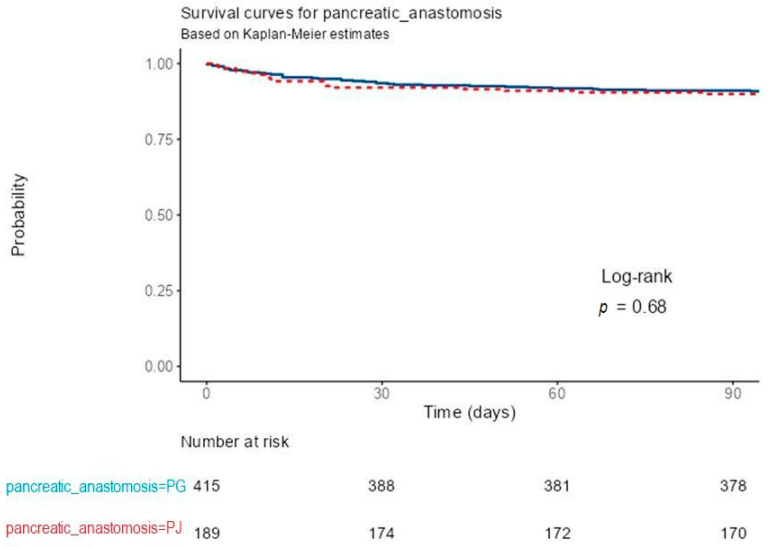
Kaplan–Meier survival curves comparing pancreaticogastrostomy (PG) and pancreaticojejunostomy (PJ) after pancreaticoduodenectomy, showing median survival among patients who died during hospitalization.

In total, 69 deaths occurred within the analyzed follow-up, 46 (11.1%) in the PG group and 23 (12.2%) in the PJ group. Cox proportional-hazards regression confirmed the absence of a statistically significant association between the type of pancreatic anastomosis and early postoperative survival. Compared with PG, PJ reconstruction was associated with a HR of 1.11 (95% CI 0.67–1.83, *p* = 0.682). Model concordance was 0.511 (standard error; SE 0.028), with an R^2^ of 0.000 and a likelihood-ratio test *p* = 0.684, indicating no predictive contribution of the anastomotic technique to survival outcomes. These findings indicate that, despite differences in perioperative complication profiles, the choice of pancreatic anastomosis did not significantly influence short-term survival (Figure 3).

## 4. Discussion

This retrospective cohort study compared the outcomes of PG and PJ following PD for periampullary tumors in a large series of 604 patients. In line with the literature, our analysis confirmed that POPF remains the most relevant and feared complication after PD, with a reported incidence between 5–30% in previous studies and persisting despite advances in surgical technique [3,4,5,15,36].

The retrospective cohort study of Mastalier et al. demonstrated that although overall outcomes between PG and PJ were comparable, PG was associated with significantly lower rates of postoperative pancreatic fistula in high-risk patients, particularly those with soft pancreatic texture, nondilated ducts, hypoalbuminemia, weight loss, or high intraoperative blood loss. Based on these predictors, the authors proposed a simplified risk score to guide individualized selection of the anastomotic technique, highlighting PG as the preferred option in patients at elevated risk of fistula [36].

Our institution’s practice pattern—favoring PG in cases with soft pancreatic remnants and small ducts, and PJ in those with firm glands and dilated ducts—reflects international consensus that reconstruction technique must be tailored to gland texture and duct size. Nevertheless, the current analysis, performed in a high-volume pancreatic surgery center, demonstrated significant differences in outcomes between PG and PJ despite these selection principles.

Specifically, the incidence of CR-POPF was significantly lower in the PG group (12.3%) compared with PJ (18.5%, *p* = 0.042). A similar trend was observed for postoperative biliary fistula (POBF: 2.9% vs. 6.3%, *p* = 0.044). Although the *p*-values for POPF (*p* = 0.042) and POBF (*p* = 0.044) indicate modest statistical significance, these findings are clinically relevant, as even small reductions in postoperative fistula rates can lead to fewer complications and shorter hospital stays. Thus, the lower incidence of POPF and POBF after PG may translate into a meaningful clinical advantage, particularly in high-risk patients with a soft pancreas or nondilated duct. These findings support several RCTs and meta-analyses that suggested PG may reduce anastomotic leakage rates compared with PJ [16,17,20,23,24]. From a physiological standpoint, PG offers a well-vascularized, thick gastric wall for anastomosis and exposes pancreatic secretions to the acidic gastric environment, potentially reducing premature enzymatic activation and local tissue injury. The retro-gastric position of the anastomosis also ensures better vascularization and mechanical protection, while the shorter distance between the pancreatic remnant and stomach may reduce anastomotic tension. Together, these factors may contribute to a more stable anastomosis and a lower risk of leakage.

In multivariable analysis, PJ, male sex, prolonged operative time, and bile contamination independently predicted clinically relevant POPF. The higher fistula rate after PJ aligns with prior evidence favouring PG, particularly in soft or small-duct glands. The association with male sex, previously observed in other cohorts, may reflect sex-related differences in gland texture or inflammatory response. Prolonged surgery and bile contamination likely indicate greater technical complexity and intra-abdominal bacterial load, both contributing to anastomotic failure. Conversely, age, vascular invasion, and blood loss were not independent predictors, underscoring the dominant influence of intraoperative and microbiological rather than baseline factors.

In the reduced multivariable model, PJ reconstruction and advanced patient age emerged as independent predictors of postoperative biliary fistula, whereas intraoperative bile culture status did not exert a significant effect. The observed association between reconstruction type and POBF mirrors the pattern identified for pancreatic fistulas, suggesting that similar technical and anatomical determinants may contribute to biliary leakage. The model’s modest explanatory capacity and limited sensitivity likely reflect the low incidence of POBF and the inherent constraints of logistic regression when applied to infrequent events. Despite these limitations, the results underscore the potential influence of reconstruction technique and patient-related factors such as frailty on postoperative biliary outcomes, emphasizing the need for confirmation in larger, prospectively designed studies.

Pancreatic texture and duct diameters were excluded from the multivariable analysis due to inconsistent and non-standardized documentation across the study period, which resulted in substantial and non-random missingness and would have introduced selection bias if included.

In a recent retrospective cohort from our institution, Moldovan et al. reported that endoscopic PBD was associated with higher rates of POPF and intra-abdominal abscess compared with surgical or no drainage, while 90-day mortality remained similar across groups [37]. Moreover, in our previous systematic review and meta-analysis on PBD in periampullary tumors, endocopic retrograde biliary drainage (ERBD) was associated with higher rates of postoperative intra-abdominal abscess and DGE compared with external drainage methods (percutaneous transhepatic biliary drainage; PTBD/ endoscopic nasobiliary drainage; ENBD), emphasizing the importance of an appropriate selection of the drainage technique according to clinical context [38]. These findings highlight how perioperative management strategies can influence postoperative morbidity without affecting short-term survival. In the present study, however, no differences were observed between the PG and PJ groups regarding the type or impact of preoperative biliary drainage, indicating that outcomes related to the anastomotic technique were not influenced by prior biliary drainage.

Importantly, other postoperative outcomes—including DGE, PPH, intra-abdominal abscess, relaparotomy, hospital stay, and 90-day mortality—were not significantly different between the groups. This suggests that, aside from fistula formation, both reconstruction methods are largely comparable in terms of global morbidity and mortality.

When placed in the context of existing evidence, our results—showing a higher rate of POPF after PJ compared with PG—contrast with the large RCTs by Bassi et al. and Keck et al., both of which demonstrated comparable fistula rates between the two reconstruction techniques [15,19]. These findings have been further supported by high-quality meta-analyses. Cheng et al. conducted a comprehensive systematic review and meta-analysis including RCTs and observational studies, and demonstrated that PG and PJ are largely comparable in terms of overall postoperative outcomes, with no clear superiority of either technique regarding CR-POPF, morbidity, or mortality [39]. Similarly, Lyu et al. (2018), in an updated meta-analysis restricted to RCTs and applying the standardized ISGPS 2016 definitions, reported no significant difference between PG and PJ with respect to the incidence of CR-POPF, PPH, or DGE [5].

In a prospective single institution matched historical control study, Wang et al. (2016) reported that the Blumgart PJ technique was associated with superior outcomes compared to PG [40]. Specifically, the incidence and severity of CR-POPF, as well as surgical mortality, were significantly lower in the Blumgart PJ group, regardless of pancreatic texture, duct size, or underlying pathology. Based on these results, the authors advocated Blumgart PJ as a rapid, straightforward, and safe option for pancreatic reconstruction following PD [40].

In our series, PPH rates did not differ significantly between the two groups compared, possibly due to surgical standardization and meticulous hemostatic technique in our center. However, our results differ from the RECOPANC trial, which reported higher rates of PPH with PG [19].

Bassi et al. (2005) reported that PG was associated with significantly fewer POBFs, postoperative collections, DGE, and a lower frequency of multiple surgical complications [15]. In contrast, our study also demonstrated a lower rate of POBF after PG, but found comparable rates of DGE and intra-abdominal abscess between the two techniques.

In the present cohort, early postoperative survival did not differ significantly between PG and PJ following PD. Both reconstruction methods achieved a 90-day survival exceeding 95%, and the median survival was undefined in each group due to the absence of a 50% event rate during follow-up. These findings suggest that the type of pancreatic anastomosis has no measurable impact on short-term mortality, which is consistent with previously published randomized and observational data. The RECOPANC trial and subsequent meta-analyses likewise demonstrated comparable early survival outcomes between PG and PJ, emphasizing that the choice of anastomotic technique primarily influences postoperative morbidity—particularly the incidence and severity of clinically relevant pancreatic fistula—rather than mortality [19]. The present results therefore reinforce the notion that both PG and PJ are safe reconstructive options in experienced hands, and that the selection of technique should be guided by surgeon expertise, intraoperative findings, and institutional preference rather than concerns regarding early postoperative survival.

Beyond confirming differences in fistula rates, our study advances current evidence by demonstrating that outcomes after pancreatic reconstruction depend not only on the anastomotic technique but also on the context of surgical practice and patient selection. By providing large real-world data from an Eastern European tertiary center, this analysis broadens the geographic and clinical applicability of prior findings and supports a risk-adapted approach, in which PG may be preferred for patients with a soft pancreas or small duct. Moreover, the lower rates of POPF and POBF observed after PG could facilitate earlier drain removal, shorter hospitalization, and smoother postoperative recovery, aligning with modern Enhanced Recovery After Surgery (ERAS) principles. These insights highlight the need for procedure standardization and stratified future trials that consider gland texture, duct size, and perioperative management rather than focusing solely on the reconstruction type.

In our center, both binding PG (Peng technique) and duct-to-mucosa PJ are performed according to standardized protocols, ensuring methodological consistency and strengthening the validity of the comparison. By applying standardized ISGPS/ISGLS definitions for POPF, DGE, PPH, and POBF, we ensured comparability with international literature.

This study has limitations inherent to its retrospective design, including potential selection bias, unmeasured confounders, and surgeon preference influencing reconstruction choice. Nevertheless, the large sample size, homogeneity of operative standards, and detailed complication reporting strengthen the validity of the findings.

Taken together, our results support the use of PG as the preferred anastomotic technique in patients at higher risk of POPF, particularly those with soft pancreas and small ducts. While PJ remains a viable option, especially in firm, dilated glands, its association with higher fistula rates in our cohort underscores the need for careful patient selection and possibly adjunctive protective measures. Future prospective, randomized studies are warranted to confirm whether PG should be recommended as the first-line reconstruction in pancreaticoduodenectomy.

## 5. Conclusions

This large single-center retrospective cohort study compared PG and PJ following PD for periampullary tumors. Our findings demonstrated that PG was associated with significantly lower rates of POPF and POBF compared with PJ, while other postoperative outcomes—including DGE, PPH, intra-abdominal abscess, relaparotomy, hospital stay, and 90-day mortality—were similar between the two groups. PJ, male sex, prolonged operative time, and bile contamination independently predicted CR-POPF, emphasizing the influence of technical complexity and intra-abdominal bacterial load on anastomotic failure. In contrast, CR-POBF was associated with PJ and advanced age, suggesting that both reconstructive technique and patient frailty contribute to biliary leakage. These findings suggest that PG appears preferable in high-risk patients, particularly those with a soft pancreatic texture or small pancreatic duct. PJ continues to be an appropriate reconstructive technique in patients presenting with a firm gland and a dilated pancreatic duct. However, both techniques remain safe and feasible when performed in experienced centers, and further prospective studies are warranted to confirm these results.

## 6. Limitations

This study has several limitations that should be acknowledged.

First, its retrospective, single-center design introduces the potential for selection bias, residual confounding, and dependence on the accuracy of institutional records. The choice of anastomotic technique was not randomized but based on intraoperative gland texture, duct size, and surgeon preference, which may have influenced postoperative outcomes.

We acknowledge that we were unable to compute texture- or duct-based risk scores (e.g., alternative Fistula Risk Score, aFRS) for the entire cohort due to incomplete and non-standardized documentation of pancreatic texture and duct diameter across the study period.

Second, although the overall cohort was large, subgroup analyses (such as patients with pylorus-preserving procedures or those requiring vascular resections) may have been underpowered to detect small differences.

Third, long-term outcomes, including exocrine and endocrine pancreatic function, quality of life, and survival, were not evaluated, limiting the conclusions to short-term perioperative results.

Fourth, the generalizability of our findings may be restricted, as all procedures were performed in a high-volume pancreatic surgery center with standardized techniques (binding PG and duct-to-mucosa PJ), which may not reflect practice in lower-volume institutions.

Finally, heterogeneity in perioperative management and unmeasured factors such as nutritional status or subtle variations in surgical technique could have influenced complication rates.

Moreover, the study represents a level III–IV grade of evidence, given its retrospective observational design, and therefore cannot establish causal relationships. External validation through prospective multicenter RCTs is essential to confirm these findings and to define the optimal reconstructive approach after PD across diverse practice settings.

## Figures and Tables

**Table 1:** Patient Demographics and Clinical Characteristics.

Variable	All Patients (n = 604)	PG (n = 415)	PJ (n = 189)	*p*-Value
Age, mean ± SD	62.3 ± 10.4	63.6 ± 9.93	62.9 ± 9.84	0.442 ***
Sex				0.565 *
Female	250 (41.4%)	175 (42.2%)	75 (39.7%)	
Male	354 (58.6%)	240 (57.8%)	114 (60.3%)	
Histology of lesion				0.358 *
Ampullary carcinoma	124 (20.5%)	94 (22.7%)	30 (15.9%)	
Benign lesions	22 (3.6%)	18 (4.3%)	4 (2.1%)	
Chronic pancreatitis	18 (3.0%)	12 (2.9%)	6 (3.2%)	
Distal cholangiocarcinoma	64 (10.6%)	47 (11.3%)	17 (9.0%)	
Duodenal carcinoma	11 (1.8%)	7 (1.7%)	4 (2.1%)	
GIST	8 (1.3%)	6 (1.4%)	2 (1.1%)	
NET	23 (3.8%)	16 (3.9%)	7 (3.7%)	
Other carcinoma sites	4 (0.7%)	3 (0.7%)	1 (0.5%)	
Pancreatic carcinoma	316 (52.3%)	205 (49.4%)	111 (58.7%)	
Premalignant lesions	14 (2.3%)	7 (1.7%)	7 (3.7%)	
Primary surgical procedure				0.008 *
PD	569 (94.2%)	398 (95.9%)	171 (90.5%)	
PPPD	35 (5.8%)	17 (4.1%)	18 (9.5%)	
Vascular invasion				0.992 **
IVC	2 (0.3%)	2 (0.5%)	0 (0.0%)	
SMV	17 (2.8%)	12 (2.9%)	5 (2.6%)	
PV	20 (3.3%)	13 (3.1%)	7 (3.7%)	
PV + SMV	3 (0.5%)	2 (0.5%)	1 (0.5%)	
no	562 (93.0%)	386 (93.0%)	176 (93.1%)	
Vascular reconstruction				1.000 **
Tangential suture	37 (90.2%)	25 (89.3%)	12 (92.3%)	
T-T anastomosis	4 (9.8%)	3 (10.7%)	1 (7.7%)	
PBD				0.701 **
Endoscopic	304 (50.3%)	204 (49.2%)	100 (52.9%)	
Percutaneous	7 (1.2%)	5 (1.2%)	2 (1.1%)	
Surgical	49 (8.1%)	32 (7.7%)	17 (9.0%)	
no	244 (40.4%)	174 (41.9%)	70 (37.0%)	
Biliculture				0.001 **
Positive	313 (51.8%)	205 (49.4%)	108 (57.1%)	
Negative	152 (25.2%)	123 (29.6%)	29 (15.3%)	
no	139 (23.0%)	87 (21.0%)	52 (27.5%)	
Operative time	282.24 ± 68.33	284.90 ± 69.39	276.40 ± 65.73	0.155 ***
Blood loss	318.0 ± 259.00	319.60 ± 247.25	314.50 ± 283.75	0.841 ***

GIST: gastrointestinal stromal tumor; IVC: inferior vena cava; NET: neuroendocrine tumor; PBD: preoperative biliary drainage; PD: pancreaticoduodenectomy; PG: pancreaticogastrostomy; PJ: pancreaticojejunostomy; PPPD: pylorus-preserving pancreaticoduodenectomy; PV: portal vein; PV-SMV: portal vein- superior mesenteric vein confluence; SD: standard deviation; SMV: superior mesenteric vein; SV: splenic vein; T-T: termino-terminal. * Chi square test. ** Fischer’s exact test. *** Student’s test.

**Table 2:** Postoperative complication rates.

Variable	All Patients (n = 604)	PG (n = 415)	PJ (n = 189)	*p*-Value
Overall complication rate	271 (44.9%)	182 (43.9%)	89 (47.1%)	0.481 *
Wound infection	28 (4.6%)	19 (4.6%)	9 (4.8%)	0.921 *
Intra-abdominal abscess	45 (7.5%)	30 (7.2%)	15 (7.9%)	0.759 *
Sepsis	17 (2.8%)	11 (2.6%)	6 (3.2%)	0.689 *
Clostridium difficile colitis	42 (7.0%)	30 (7.2%)	12 (6.3%)	0.693 *
Pulmonary complications	37 (6.1%)	27 (6.5%)	10 (5.3%)	0.564 *
Cardiovascular complications	35 (5.8%)	26 (6.3%)	9 (4.8%)	0.463 *
PPAP	23 (3.8%)	17 (4.1%)	6 (3.2%)	0.583 *
PPH	89 (14.7%)	65 (15.7%)	24 (12.7%)	0.341 *
Lymph leakage	12 (2.0%)	6 (1.4%)	6 (3.2%)	0.158 *
POPF	86 (14.2%)	51 (12.3%)	35 (18.5%)	**0.042** *
POBF	24 (4.0%)	12 (2.9%)	12 (6.3%)	**0.044** *
Gastrojejunostomy leakage	7 (1.2%)	3 (0.7%)	4 (2.1%)	0.138 *
DGE	44 (7.3%)	30 (7.2%)	14 (7.4%)	0.938 *
Celiac axis ischemia	7 (1.2%)	4 (1.0%)	3 (1.6%)	0.507 *
Mesenteric infarction	19 (3.1%)	14 (3.4%)	5 (2.6%)	0.635 *
Relaparotomy	45 (7.5%)	33 (8.0%)	12 (6.3%)	0.487 *
Postoperative hospital stay, mean ± SD	15.02 ± 10.84	14.60 ± 10.96	16.00 ± 10.52	0.135 **
90-day mortality	56 (9.27%)	37 (8.91%)	19 (10.05%)	0.768 *

DGE: Delayed gastric emptying; PBD: Preoperative biliary drainage; POBF: Postoperative biliary fistula; POPF: Postoperative pancreatic fistula; PPAP: Postpancreatectomy acute pancreatitis; PPH: Post-pancreatectomy hemorrhage SD: standard deviation. * Chi square test. ** Student’s test.

**Table 3:** Univariate and multivariate logistic regression for risk factors associated with postoperative pancreatic fistula (POPF).

Predictor	Unadjusted OR (95% CI)	*p* (Univariate)	Adjusted OR (95% CI)	*p* (Multivariate)
Pancreatic anastomosis (PJ vs. PG)	3.39 (1.92–5.98)	**0.00002**	3.31 (1.79–6.13)	**0.0001**
Sex (male vs. female)	3.79 (1.91–7.54)	**0.00014**	3.83 (1.88–7.80)	**0.0002**
Age (years)	1.01 (0.97–1.03)	0.99	1.02 (0.98–1.05)	0.340
Pancreatectomy type (PPPD vs. PD)	2.61 (0.87–7.78)	0.086	2.41 (0.71–8.25)	0.161
Vascular invasion (yes vs. no)	1.09 (0.40–2.98)	0.85	1.09 (0.37–3.26)	0.874
Operative time > median (280 min)	1.52 (0.87–2.63)	0.14	2.01 (1.07–3.81)	**0.031**
Blood loss > median (250 mL)	0.97 (0.56–1.67)	0.91	0.88 (0.47–1.64)	0.677
Positive bile culture (yes vs. no)	2.74 (1.34–5.59)	0.0057	2.13 (1.00–4.53)	**0.049**

PD: pancreaticoduodenectomy; PG: pancreaticogastrostomy; PJ: pancreaticojejunostomy; PPPD: pylorus-preserving pancreaticoduodenectomy. Model performance: χ^2^(8) = 45.6, *p* < 0.001; Nagelkerke R^2^ = 0.19; AUC = 0.77; overall accuracy = 85.2%; specificity = 98.5%; sensitivity = 13.1%.

**Table 4:** Multivariable logistic regression for risk factors associated with postoperative biliary fistula (POBF).

Predictor	Unadjusted OR (95% CI)	*p* (Univariate)	Adjusted OR (95% CI)	*p* (Multivariate)
Pancreatic anastomosis (PJ vs. PG)	3.14 (1.21–8.16)	**0.018**	3.20 (1.21–8.44)	**0.019**
Age (years)	1.05 (1.00–1.10)	0.058	1.06 (1.00–1.13)	**0.046**
Positive bile culture (yes vs. no)	1.73 (0.56–5.35)	0.339	1.39 (0.44–4.41)	0.573

CI: confidence interval; OR: odds ratio; PG: pancreaticogastrostomy; PJ: pancreaticojejunostomy. Model performance: χ^2^(3) = 10.34, *p* = 0.016; Nagelkerke R^2^ = 0.079; AUC = 0.71; overall accuracy = 96.1%; specificity = 100%; sensitivity = 0%.

## Data Availability

The original contributions presented in this study are included in the article. Further inquiries can be directed to the corresponding author(s).

## Supplementary Materials

The following supporting information can be downloaded at: https://www.mdpi.com/article/10.3390/medicina61112051/s1, Table S1: STROBE_checklist_cohort.

## Abbreviations

The following abbreviations are used in this manuscript:

aFRS	alternative Fistula Risk Score
aOR	adjusted Odds ratio
AUC	Area under ROC curve
BL	Biochemical leak
CBD	Common bile duct
CCI	Comprehensive Complication Index
CI	Confidence interval
CR-POBF	Clinically relevant postoperative biliary fistula
CR-POPF	Clinically relevant postoperative pancreatic fistula
CT	Computed tomography
DGE	Delayed gastric emptying
ERBD	Endoscopic retrograde biliary drainage
ENBD	Endoscopic nasobiliary drainage
ERAS	Enhanced Recovery After Surgery
GI	Gastrointestinal
GIST	Gastrointestinal stromal tumor
HPB	Hepatopancreatobiliary
HR	Hazard ratio
IVC	Inferior vena cava
ISGPF	International Study Group on Pancreatic Fistula
ISGLS	International Study Group of Liver Surgery
ISGPS	International Study Group of Pancreatic Surgery
NCCN	National Comprehensive Cancer Network
NET	Neuroendocrine tumor
OR	Odds ratio
PBD	Preoperative biliary drainage
PD	Pancreaticoduodenectomy
PG	Pancreaticogastrostomy
PJ	Pancreaticojejunostomy
POBF	Postoperative biliary fistula
POH	Postoperative hyperamylasemia
PPAP	Postpancreatectomy acute pancreatitis
PPH	Post-pancreatectomy hemorrhage
PPPD	Pylorus-preserving pancreaticoduodenectomy
PTBD	Percutaneous transhepatic biliary drainage
PV	Portal vein
PV-SMV	Portal vein-superior mesenteric vein confluence
RCT	Randomized controlled trial
SD	Standard deviation
SE	Standard error
SMV	Superior mesenteric vein
SSI	Surgical site infection
STROBE	STrengthening the Reporting of OBservational studies in Epidemiology
SV	Splenic vein
T-T	Termino-terminal
